# A patient decision aid for breast cancer patients deciding on their radiation treatment, no change in decisional conflict but better informed choices

**DOI:** 10.1016/j.tipsro.2021.08.002

**Published:** 2021-08-29

**Authors:** D.B. Raphael, N.S. Russell, B. Winkens, J.M. Immink, P.G. Westhoff, M.C. Stenfert Kroese, M.R. Stam, N. Bijker, C.M.J. van Gestel, T. van der Weijden, L.J. Boersma

**Affiliations:** a Department of Radiation Oncology (Maastro), GROW School for Oncology and Developmental Biology, Maastricht University Medical Centre+, Maastricht, the Netherlands; b Department of Family Medicine, CAPHRI Care and Public Health Research Institute, Maastricht University, Maastricht, the Netherlands; c Department of Radiotherapy, Netherlands Cancer Institute, Antoni van Leeuwenhoek, Amsterdam, the Netherlands; d Department of Methodology and Statistics, CAPHRI Care and Public Health Research Institute,Maastricht University, Maastricht, the Netherlands; e Department of Radiation Oncology, Reinier de Graaf Hospital, Delft, the Netherlands; f Department of Radiation Oncology, Leiden University Medical Center, Leiden, the Netherlands; g Department of Radiation Oncology, Radboud University Medical Center, Nijmegen, the Netherlands; h Radiotherapy Group, Deventer, the Netherlands; i Radiotherapy Group, Arnhem, the Netherlands; j Department of Radiation Oncology, Amsterdam University Medical Centers, the Netherlands; k Zuidwest Radiotherapeutisch Instituut (ZRTI), the Netherlands

## Abstract

**Background and Purpose:**

In selected breast cancer patients, radiation treatment (RT) lowers the recurrence risk, with minor or no improvement of survival. In these patients, the choice to undergo RT is considered a preference-sensitive decision. To facilitate shared decision-making (SDM) for this choice, a patient decision aid was made. We aimed to evaluate the effect of the PtDA on decisional conflict.

**Material and methods:**

We performed a multi-center pre- and post-intervention study (BRASA-trial). The first 214 patients made a choice without support of the PtDA; the subsequent 189 patients received a link to the PtDA. The primary endpoint was decisional conflict; secondary endpoints were perceived SDM and knowledge on treatment options. Patients filled out questionnaires immediately after, and three months after their decision. Data were analyzed with multi-level regression analysis.

**Results:**

After correcting for the difference in age and educational level, the mean (±SD) decisional conflict for the intervention group (27.3 ± 11.4) was similar to the control group (26.8 ± 11.4; difference = 0.86, 95 %CI 1.67,3.36) three months after their decision. This also applied to perceived SDM. Patients exposed to the PtDA pursued additional treatment less often (45% vs 56%, odds ratio 0.59, 95 %CI 0.37,0.95) and scored significantly higher on the knowledge test (7.4 ± 2.5 vs 6.1 ± 2.7, corrected difference = 1.0, 95 %CI 0.50,1.49). There was no significant increase in consultation time.

**Conclusions:**

Handing out the PtDA was not associated with improved scores in decisional conflict or perceived SDM, but it was associated with a choice for less additional treatment and better knowledge about the treatment options.

## Highlights

• No difference was found in decisional conflict with or without PtDA use.
• Patients who used the PtDA had more knowledge, suggesting a better informed choice.
• Patients who used the PtDA chose more often for a less intensive treatment.
• The use of the PtDA was not significant associated with a longer consultation time.

## Introduction

In the process of shared decision-making (SDM), patients and clinicians collaborate to select the treatment that fits the patient best [1], [2]. The patient knows her own personal situation, values, and preferences best, whereas the clinician has the most knowledge about her medical situation. These aspects need to be elicited to achieve optimal sharing of the decision-making process. Patient decision aids (PtDAs) are tools that support the SDM process.

[3], [4]. When PtDAs are used, patients are more satisfied with the decision made. In addition, they have more knowledge on their treatment options, are less likely to opt for more additional treatment, and feel more engaged in SDM [3].

The decision on whether radiotherapy (RT) is offered is usually made according to international and national guidelines. In certain settings, however, administration of post-operative RT can be considered a preference-sensitive decision. In these situations, there is evidence that RT reduces local recurrence risk, with a risk of causing side effects, but with no or uncertain benefit to long-term survival [5], [6], [7], [8], [9], [10], [11], [12], [13], [14], [15]. In these situations, guidelines may recommend discussing the treatment of choice with the patient [16], or this may be decided by the multidisciplinary team. The latter might happen when guidelines do give a clear recommendation to offer RT, but the latest literature suggests a lower benefit.

There are a number of known preference-sensitive situations when choosing for breast cancer RT, such as patients with a low to intermediate risk on local recurrence after mastectomy, older patients undergoing breast-conserving therapy (BCT) for low-risk invasive breast cancer, the indication for boost irradiation to the tumor bed in case of BCT, or patients with low-risk Ductal Carcinoma In Situ (DCIS) undergoing BCT [9], [17], [18], [19], [20]. To support SDM in these preference-sensitive treatment decisions, we developed an online PtDA [13], [14] according to the international (IPDAS) guidelines [21], [22], [23], [24]. The PtDA starts with an introduction on SDM, and points out that there is a choice to be made. It explains how RT is performed in text and in an animation film. The PtDA gives information on the possible effect and side effects of RT. Additionally it elicits the patients’ preferences. In a review by Vromans et al. this PtDA scored 83 out of 100 points [25]. The PtDA is available online in Dutch - with an additional English translation - at www.beslissamen.nl. We evaluated the PtDA in a multi-centre pre- and post-intervention study in 13 out of the 19 RT centers in the Netherlands [26]. The aim of this study is to investigate whether the PtDA resulted in improved decisional quality, an increased perceived level of SDM, and improved knowledge on the different treatment options. In addition, we investigated the impact of the PtDA on the choice for more or less (additional) radiation treatment, as well as its impact on consultation length.

## Materials and methods

### Study design

We performed a multi-center pre- and post-intervention study. In the pre-intervention group, i.e., the control arm, patients were included before introducing the PtDA. In the post-intervention group, i.e., the intervention arm, patients were offered the PtDA.

### Study population

We included patients if they were 18 years or older, had a breast cancer diagnosis, and were sufficiently able to understand written Dutch to use the PtDA. All patients were faced with a preference-sensitive decision on RT, according to the multi-disciplinary team (MDT) of their treating hospital or their treating clinician. Patients had to fit in one of the four pathways the PtDA was developed for: boost/no-boost group, chest wall RT group, low-risk breast cancer group and DCIS group (appendix A). All patients were included in the trial by their radiation oncologist.

### Intervention

Once recruitment of the control arm was complete, clinicians were instructed on how to use the PtDA. We provided an e-learning opportunity, but this training was not obligatory. The logistics for referring patients to the PtDA were adjusted to the existing logistics and referral systems of the participating centers [26]. Ideally, patients were identified in the MDT. Patients received the PtDA-link from the surgery department (*n* = 33) or from the RT department (*n* = 135), this could either be during the consultation or previous to the consultation through regular mail. Ten patients received the PtDA link through another route. For ten patients, it was unknown via which route they received the link to the PtDA.

### Assessments

Patients were requested to complete questionnaires within three days after they had made their decision (T1). After three months (T2), the research team sent the follow-up questionnaire by mail or e-mail, according to the patient’s preference (Table 1). We also recorded the final treatment decision concerning the RT. The participating clinicians were asked to fill out a Case Report Form including medical information (tumor type and treatment characteristics) and consultation length immediately after the post-operative consultation.

**Table 1:** Overview of the time schedule for data acquisition via patient questionnaires and via Case Report Forms (CRFs) filled out by clinicians. T1 is < 3 days after the decision, T2 is 3 months after the decision.

	**Type of data**	**T1**	**T2**
**Patients**	Decisional Conflict Scale	x	x
	SDM-Q9	x	
	CollaboRATE	x	
	Patient knowledge	x	
	Patient preferences	x	x
	Treatment chosen	x	

**Clinicians**	CRF with disease and treatment characteristics, and consultation length	x	

### Outcomes

The primary endpoint was decisional conflict at three months after the decision had been made, measured using the Decisional Conflict Scale (DCS) (Appendix B). The DCS measures how certain patients are about their decision and how they feel about the decision-making process. A validated Dutch translation was used [27], [28].The DCS consists of 16 questions evaluated on a 5-point Likert scale (scoring 0–4). Higher scores imply that more decisional conflict is experienced.

The secondary outcomes were perceived level of SDM and patient knowledge on their treatment options, measured < 3 days after deciding on RT. Perceived level of SDM was measured with the SDM-Q9 (Appendix C) [29] and the CollaboRATE (Appendix D)[30]. The SDM-Q9 consist of nine questions, evaluated on a 6-point Likert scale (scoring 0–5). CollaboRATE consist of three questions evaluated on a 10-point Likert scale [31], [32]. On both questionnaires, a higher score expresses a higher level of experienced SDM. These are both validated questionnaires of which Dutch translations were used, available on the websites of the questionnaires developers [30], [33]. Patient knowledge was measured by a knowledge test developed by our research team, in the absence of a validated test. This questionnaire consisted of 11 questions aligned to the content of the PtDA ensuring high content validity. Patients scored one point for each correct answer. One point was deducted for each incorrect answer; no points were given if the patient did not know the answer (appendix E) [34].

To investigate important attributes for the decision-making process, we developed a questionnaire consisting of nine statements on different attributes in the decision-making process, as well as one question asking to prioritize which three attributes were the most important for the decision made (appendix F). These statements are similar to the statements asked for in the PtDA, which was developed together with patients [13]. All self-developed questionnaires were pilot tested on comprehension and difficulty on breast cancer patients prior to the study.

### Sample size calculation

Considering an effect size on the decisional conflict scale of 0.30–0.40 as a meaningful difference (28,35], we aimed to demonstrate an effect size (if present) of 0.40, with a power of 80% (*Z* = 0.84) and a two-sided alpha of 0.05 (*Z* = 1.96). This required 99 patients per group. Because the decision-making process is influenced by input of the individual clinician, each clinician was considered a different cluster. Assuming each clinician would include 6 patients on average and intra-class correlation equals 0.04 [36], the design effect (=1 + (6–1)*0.04) equals 1.2. Accounting for an (additional) 10% loss in efficiency due to unequal cluster sizes [37], the required sample size per group was calculated to be equal to 99*1.2/0.9 = 132 patients within 22 clinicians. Accounting for 20% dropout, 28 recruiting clinicians (28clusters) per group were required, resulting in 168 patients per group.

### Data analyses

Patient and treatment characteristics, as well as patient preferences, were described using the mean value (standard deviation, SD) for numerical variables and number of patients per category (%) for categorical ones. Differences in these characteristics between control and intervention groups were assessed using Chi-square test or fisher exact test where appropriate for categorical variables, while independent-samples *t*-test was used for numerical variables. For the DCS, the SDM-Q9, and the CollaboRATE, we calculated the sum score for the questionnaire according to the manuals of these questionnaires [31], [35], [38]. We used linear mixed models to analyze differences in the scores on the questionnaires between the control and the intervention group. A random intercept on the clinician level accounted for the clustering of patients within a clinician. In addition to the treatment group, we also included characteristics that differed significantly and/or substantially between the groups in the fixed part of the model to adjust for potential confounding. For the outcome measure of treatment choice, the same variables were included in a generalized linear mixed model with a logit link function to account for the binary data. We did not impute missing outcome, as this was accounted for in the mixed model analysis due to its likelihood-based approach, assuming that outcome data were missing at random (MAR). All analyses were performed using IBM SPSS Statistics for Windows (version 25; Armonk, NY, USA, IBM Corp.), except for mixed model analyses, which were performed using STATA (StataCorp. 2009. *Stata Statistical Software: Release 11*. College Station, TX: StataCorp LP.). A two-sided *p*-value ≤ 0.05 was considered statistically significant.

### Ethical standards

The trial was approved by the institutional review board of Maastro and the Netherlands Cancer Institute and was carried out in accordance with the declaration of Helsinki. All participants gave informed consent after reading written trial information. Patients were allocated a study code without personal identifiers.

## Results

**Fig. 1:**
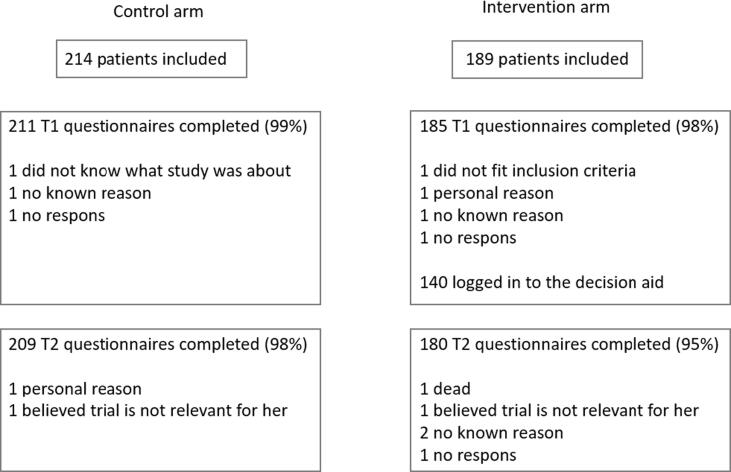
Flow diagram with an overview of included patients per arm.

As described in a prior publication [26], patients were recruited from 13 out of 19 radiation oncology centers in the Netherlands. Between October 2017 and October 2018, 214 patients were included in the control arm, of which 211 filled in the T1 questionnaire and 209 the T2 questionnaire. Between October 2018 and July 2019, 189 patients were included in the intervention arm, of which 185 filled in the T1 questionnaire, 140 patients used the PtDA [26], and 180 filled in the T2 questionnaire (Fig. 1). In total 104 different clinicians included patients in the study. Of these 104 clinicians, 65 included patients in the control arm and 76 in the intervention arm.

The mean age was 60.4 (*SD* = 11.3) years in the intervention arm and 62.8 (*SD* = 12.6) years in the control arm (*p* = 0.050). In the intervention arm, 28% of the patients had a low educational level, 32% had middle education, and 40% was highly educated; in the control arm, this was 42%, 30%, and 28% respectively (p < 0.007). Disease and treatment characteristics were comparable between both groups (Table 2).

**Table 2:** Patient characteristics per study-arm.

		Intervention (*n* = 189)	Control *(n* = 214)	*p*-value
Mean Age (in years)		60.4 (*SD* = 11.3)	62.8 (*SD* = 12.6)	0.050
Indication for SDM on (additional) RT yes or no:	DCIS Low-risk invasive Boost Chest wall Missing	62 (33%) 58 (31%) 47 (25%) 21 (11%) 1	64 (30%) 65 (30%) 62 (29%) 23 (11%) 0	0.824

Educational level	Low Middle High Missing	50 (28%) 59 (32%) 73 (40%) 7	86 (42%) 63 (30%) 58 (28%) 7	0.007

SDM indicated in MDT	No Yes Missing	62 (34%) 120 (66%) 7	90 (44%) 114 (56%) 10	0.044

Axillary treatment	Sentinel node procedure Axillary lymph node dissection MARI Sentinel node procedure + MARI Sentinel node procedure + RT Axillary lymph node dissection + MARI + RT Sentinel node procedure + Axillary lymph node dissection + MARI + RT None Missing	109 (58%) 5 (23%) 2 (1%) 2 (1%) 4 (2%) 0 0 65 (34.8%) 2	137 (65%) 6 (3%) 3 (1%) 1 (0.5%) 2 (1%) 1 (0.5%) 1 (0.5%) 61 (28.5%) 2	0.694*

Systemic therapy	Yes No Missing	53 (28%) 134 (71%) 2	67 (31%) 147 (69%) 0	0.458

Breast surgery	Breast-conserving surgery Amputation with direct reconstruction Amputation with delayed reconstruction Amputation without reconstruction Amputation, reconstruction unknown Missing	167 (89%) 12 (6%) 2 (1%) 4 (2%) 3 (2%) 1	191 (89%) 9 (4%) 3 (1%) 7 (3%) 4 (2%) 0	0.833*

cTNM	T0 T1 T2 T3 Missing N0 N1 Missing	66 (35%) 96 (51%) 23 (12%) 3 (2%) 1 179 (95%) 9 (5%) 1	75 (36%) 97 (46%) 35 (17%) 3 (1%) 4 197 (94%) 12 (6%) 5	0.601* 0.671

pTNM	T0 T1 T2 T3 Missing N0 N1 Missing	86 (46%) 92 (49%) 9 (5%) 0 2 172 (92%) 15 (8%) 2	86 (41%) 103 (49%) 22 (10%) 0 3 185 (89%) 24 (11%) 5	0.098 0.248

Histology	DCIS Invasive, no specific type (NST) Invasive lobular carcinoma Other Missing	62 (33%) 106 (56%) 11 (6%) 9 (5%) 0	68 (32%) 119 (56%) 16 (8%) 9 (4%) 2	0.684

Bloom-Richardson tumor grade	1 2 3 Missing	61 (34%) 80 (44%) 41 (23%) 7	72 (35%) 86 (42%) 46 (23%) 10	0.923

Receptor status	ER - ER + Missing PR - PR + Missing HER2 - HER2 + Missing	83 (44%) 105 (56%) 1 98 (52%) 90 (48%) 1 182 (97%) 6 (3%) 1	87 (41%) 126 (59%) 1 109 (51%) 104 (49%) 1 199 (93%) 14 (7%) 1	0.515 0.632 0.193

*Fisher’s exact test.

MDT = multi-disciplinary team, RT = radiotherapy, MARI = MARI method (marking of the axilla with radioactive iodine seeds).

We corrected for baseline characteristics that differed between both groups (age and educational level) in all regression analyses. There was no significant difference between the intervention and control arm in the primary endpoint, i.e. DCS at 3 months after the decision, (27.3 (SD 12.9) vs mean 26.8 (SD 11.4) (p = 0.510)) (Table 3). In addition, no significant difference was found in measures < 3 days after the decision (T1): DCS (mean 27.3 (SD 12.9) vs 26.2 (SD 12.4) (p = 0.412)), and perceived SDM level measured with the CollaboRATE (mean 88.6 (SD 14.4) vs 88.9 (SD 15.8) (p = 0.919)) and the SDM-Q9 (mean 74.0 (SD 19.7) vs 72.2 (SD 22.4) (p = 0.418)). Patients in the intervention arm scored better on the knowledge test (mean 7.4 (SD 2.5) vs 6.1 (SD 2.7) (p < 0.001)) and chose less often for (additional) RT compared to the control group (44.5% vs 55.7% (OR 0.59 (95 %CI 0.37–0.95)). No significant difference in consultation length (41.7 min vs 40.8 min (p = 0.276)) and number of consultations needed to make the decision (2.22 vs 2.01 (p = 0.869)) was seen between the intervention and control arm.

**Table 3:** Results of primary and secondary outcome measures. Raw data are summarized with absolute scores and numbers. The *p*-values represent the results of the linear and logistic mixed model analyses, corrected for age and educational level. T1 is < 3 days after the decision, T2 is 3 months after the decision.

Outcome measure	Intervention Mean (SD) N = 189	Control Mean (SD) N = 214	Corrected difference between intervention and control (95% CI)*
T2 DCS (0–100) Missing	27.3 (12.9) 14	26.8 (11.4) 7	0.85 (-1.67, 3.36)



T1 DCS (0–100) Missing	27.3 (12.9) 8	26.2 (12.4) 8	1.10 (-1.52, 3.72)

T1 Knowledge (-11–11) Missing	7.4 (2.5) 10	6.1 (2.7) 9	1.00 (0.50, 1.49)

T1 CollaboRATE (0–100) Missing	88.6 (14.4) 4	88.9 (15.8) 6	−0.16 (-3.32, 2.99)

T1 SDMQ9 (0–100) Missing	74.0 (19.7) 6	72.2 (22.4) 8	1.81 (-2.56, 6.18)

Consultation length (min) Missing	41.7 (13.5) 19	40.8 (14.3) 27	1.52 (-1.21, 4.25)

Number of consultations Missing	2.22 (7.2) 4	2.01 (6.78) 3	0.02 (-0.20, 0.24)



	Intervention	Control	OR (95 %CI)

More (additional) treatment chosen (%) Missing	44.52 7	55.7 4	0.59 (0.37, 0.95)

*Corrected for age and educational level.

The important attributes for the decision-making process were similar for the intervention and control arm (appendix F). The most important attributes, both at T1 and T2, were the local recurrence risk, the advice of the clinician, and the fact that choosing to undergo RT can give peace of mind. The least frequently chosen attributes were the cosmetic results and the daily trip to the RT center.

## Discussion

We have shown that the patient decision aid (PtDA) was not significantly associated with a better score on the decisional conflict scale (DCS), neither immediately after the decision was made nor three months later. We also found no statistically significant difference in the experienced level of shared decision making (SDM) and consultation length. We did find that patients to whom the link to the PtDA had been provided (the intervention group) chose less often for (additional) radiation treatment (RT) and that they had improved scores in the knowledge test.

The findings on DCS and SDM were disappointing, because the aim of a PtDA is to support SDM. SDM in turn aims to ensure that patients chose the treatment that corresponds best with the patients’ situation and preferences. Several instruments have been developed to measure decisional quality, but no instrument covers all aspects [39]. Since the DCS is frequently used to measure the effect of PtDAs, and Stacey et al. showed that DCS decreased after PtDA use, we chose DCS as our primary outcome [4], [39], [40]. Recently, however, Garvelink et al. [45] showed that it may also matter at what time-point the DCS is applied. They found a significant difference in DCS after PtDA use shortly (1–3 months) after the decision was made, but this difference was not significant immediately (<1 month) after the decision was made or in the long term [41]. We found scores varying around 26–27 both immediately and three months after the decision. A score on the DCS of > 25 is considered to be elevated decisional conflict [35], and our scores are only slightly above this cut-off. This raises the question of how much effect we could expect from the PtDA. Others argued that DCS might even rise immediately after PtDA use, because patients might be more aware that there is a difficult decision to be made [42].

We also aimed to measure the process of SDM. For this outcome, several questionnaires are available, but there are no high-quality tools to measure perceived SDM [43], [44], and the available tools are all hampered by a strong ceiling effect [45], [46]. We also found relatively high scores on the SDM-Q9 (*M* = 72.2 and 74.0) compared to the review of Doherr et al. (mean scores 25–75) and CollaboRATE (*M* = 88.9 and 88.6). This might suggest that the SDM process of the participating clinicians was already relatively good, so that there was not enough room for improvement [31], [46]. However, a high pre-trial level of SDM behavior may be unlikely, as many papers show that there is generally much room for improvement in this area, and we did not provide extensive training on SDM [47].

We found that patients who received the PtDA made different choices, compared to patients who did not receive the PtDA: they chose less often for (additional) RT. This suggests that the PtDA had an added effect on the deliberation of patients deciding on (additional) RT. Although patients indicated that they considered recurrence risk and peace of mind of choosing for RT as important attributes, patients in the intervention arm were more likely to decide to refrain from (additional) RT. We hypothesize they might be more aware of the limited gain of (additional) RT after using the PtDA since we also found improved scores on the knowledge test [4].

We found no difference in consultation length and number of consultations for the patients who received the PtDA. This may stimulate implementation, since it is known that clinicians fear that SDM and the use of PtDAs may consume too much time and is a frequently mentioned barrier for implementation of PtDAs [48], [49].

A limitation in our study is the lack of randomization. We chose to perform a pre- and post-intervention study, because there is currently a momentum for developing tools to incorporate SDM in clinical practice in the Netherlands [50] and we expected that hospitals would not accept randomization. Clinicians who are instructed how to work with the PtDA might already change their communication style and we therefore only performed an intention to treat analysis. Also, many hospitals were represented in the process of developing the PtDA; therefore, randomization at hospital level was not possible. Since 13 different centers included patients, numbers per center were too small to run subgroup analyses on the different centers. The drawback of our approach may be that participating clinicians might have changed their information provision on the different treatment options over time, independently of the PtDA use, particularly because there is a movement towards treatment de-escalation in breast cancer care [51], [52], [53]. Also, patients in the intervention arm were slightly younger and were more highly educated than patients in the control arm, suggesting a selection bias. We did correct for these inequalities in our analysis, which improved reliability of our results. A strength of our approach is that we were able to show the effect of the PtDA in realistic setting of daily clinical practice [54], [55]. We had more referring clinicians than required for our sample, which increases the statistical power of the study.

Although we did not find a significant effect on the perceived level of SDM, the additional value of the PtDA was evaluated by patients as “good”. Of the patients who used the PtDA, 88% considered the PtDA to be useful for the decision-making process [26]. Therefore, more research is needed on how to improve integration of the PtDA in clinical practice while simultaneously improving the SDM process overall [56], [57].

## Conclusion

We found no significant improvement on the DCS or on perceived level of SDM after handing out the PtDA. However, we did find that patients to whom the PtDA was provided, more often chose to refrain from (additional) RT, and showed better knowledge about the different treatment options, without using additional consultation time.

## Declaration of Competing Interest

The authors declare that they have no known competing financial interests or personal relationships that could have appeared to influence the work reported in this paper.

## Figures and Tables

**Table:** 

Patients with low-risk ductal carcinoma in situ (DCIS) after breast-conserving surgery deciding on whole/partial breast RT or no RT (DCIS group).
Patients with low-risk invasive ductal carcinoma after breast-conserving surgery deciding on whole/partial breast RT or no RT (low-risk breast cancer group).
Patients with intermediate-risk breast cancer after mastectomy deciding on thoracic wall RT or no RT (chest wall irradiation group).
Patients with intermediate-risk breast cancer after breast-conserving surgery deciding on whole breast RT with or without an extra boost dose to the tumor bed (boost/no-boost group).

**Table:** 

	**Strongly Agree**	**Agree**	**Neither Agree Nor Disagree**	**Disagree**	**Strongly Disagree**
**I know which options are available to me.**					
**I know the benefit of each option.**					
**I know the risks and side effects of each option.**					
**I am clear about which benefits matter most to me.**					
**I am clear about which risks and side effects matter most to me.**					
**I am clear about which is more important to me (the benefits or the risks and side effects).**					
**I have enough support from others to make a choice.**					
**I am choosing without pressure from others.**					
**I have enough advice to make a choice.**					
**I am clear about the best choice for me.**					
**I feel sure about what to choose.**					
**This decision is easy for me to make.**					
**I feel I have made an informed choice.**					
**My decision shows what is important to me.**					
**I expect to stick with my decision.**					
**I am satisfied with my decision.**					

**Table:** 

	**Completely disagree**	**Strongly disagree**	**Somewhat disagree**	**Somewhat agree**	**Strongly agree**	**Completely agree**
**My doctor made clear that a decision needs to be made.**						
**My doctor wanted to know exactly how I want to be involved in making the decision.**						
**My doctor told me that there are different options for treating my medical condition.**						
**My doctor precisely explained the advantages and disadvantages of the treatment options.**						
**My doctor helped me understand all the information.**						
**My doctor asked me which treatment I prefer.**						
**My doctor and I thoroughly weighted the different treatment options.**						
**My doctor and I selected a treatment option together.**						
**My doctor and I reached an agreement on how to proceed.**						

**Table:** 

**How much effort was made to help you understand your health issue?**									
**0 no effect**	**1**	**2**	**3**	**4**	**5**	**6**	**7**	**8**	**9 every effort was made**

**How much effort was made to listen to the things that matter most to you about your health issues?**									
**0 no effect**	**1**	**2**	**3**	**4**	**5**	**6**	**7**	**8**	**9 every effort was made**

**How much effort was made to include what matters most to you in choosing what to do next?**									
**0 no effect**	**1**	**2**	**3**	**4**	**5**	**6**	**7**	**8**	**9 every effort was made**

**Table:** 

You received a lot of information about the advantages and disadvantages of choosing for radiotherapy or to leave radiotherapy out of your treatment. Here you see some questions about this subject. Please answer the questions with the knowledge you have, do not look up the right answers.			
	True	False	Don’t know
The radiation treatment, including the time to get dressed and undressed, takes 30 min per day.			
For the radiation treatment I have to go to the hospital on a daily basis, only on the weekdays.			
I will feel pain during the radiation treatment procedure.			
During the radiation treatment I will be radioactive and I will not be allowed to come close to children.			
If the surgeon removed the tumor completely, radiation treatment is not necessary.			
The shape and the sensitivity of my breast can change even years after the radiation treatment.			
It is likely that I will feel nauseous as a consequence of the radiation treatment.			
The skin reaction can worsen after the radiation treatment before it gets better.			
Due to the radiation treatment, my breast might stay painful in the long term.			
Due to the radiation treatment, I might feel tired for some weeks.			

**Table:** 

These are statements about the decision to undergo radiotherapy or to leave radiotherapy out of your treatment. We would like to ask you to read these statements and to thing about the decision you made. Please indicate whether you agree or disagree with this quote. This way, you indicate which attributes were important to you when making the decision.				
	T1		T2	
	Control (%)	Intervention (%)	Control (%)	Intervention (%)
1. I think the idea of radiation is really awful Fully agree Agree Do not agree/disagree Disagree Fully disagree Missing	13 (6) 72 (34) 54 (26) 56 (27) 15 (7) 0	17 (9) 60 (32) 42 (23)48 (26) 16 (9) 2	15 (7)64 (31) 51 (25) 54 (26) 23 (11) 7	10 (6) 57 (32) 48 (27) 50 (28) 14 (8) 10
2. I’ll do whatever it takes to reduce the chances of tumor recurring Fully agree Agree Do not agree/disagree Disagree Fully disagree Missing	88 (42) 79 (38) 21 (10) 18 (9) 4 (2) 0	70 (39) 61 (34) 27 (15) 21 (12) 1 (0.6) 5	82 (39) 88 (42) 25 (12) 13 (6) 1 (0.5) 5	62 (35) 74 (41) 26 (15) 16 (9) 1 (0.6) 10
3. I’m put off by the idea of daily radiation treatment/extra days of radiotherapy (boost) Fully agree Agree Do not agree/disagree Disagree Fully disagree Missing	7 (3) 69 (33) 51 (24) 64 (31) 18 (9) 1	9 (5) 41 (23) 44 (25) 64 (36) 21 (12) 6	11 (5) 68 (33) 42 (20) 69 (33) 19 (9) 5	5 (3) 49 (27) 49 (27) 55 (31) 21 (12) 10
4. I’m put off by the radiation side effects Fully agree Agree Do not agree/disagree Disagree Fully disagree Missing	33 (16) 88 (42) 40 (19) 41 (20) 7 (3) 1	39 (22) 80 (44) 36 (20) 21 (12) 5 (3) 4	32 (15) 81 (39) 42 (20) 43 (21) 11 (5) 5	27 (15) 77 (43) 38 (21) 31 (17) 6 (3) 10
5. The cosmetic result in the long term is important to me Fully agree Agree Do not agree/disagree Disagree Fully disagree Missing	14 (7) 42 (20) 62 (30) 67 (32) 25 (12) 0	13 (7) 57 (32) 51 (28) 47 (26) 12 (7) 5	13 (6) 45 (22) 62 (30) 63 (30) 25 (12) 6	7 (4) 49 (28) 61 (34) 48 (27) 13 (7) 11
6. Choosing for radiotherapy/extra radiotherapy (boost) gives me peace of mind Fully agree Agree Do not agree/disagree Disagree Fully disagree Missing	31 (15) 66 (31) 39 (19) 57 (27) 17 (8) 0	27 (15) 46 (25) 36 (20) 54 (30) 19 (10) 3	28 (13) 72 (34) 47 (22) 48 (23) 14 (7) 5	23 (13) 52 (29) 36 (20) 48 (27) 20 (11) 10
7. What my health care provider advises is important to make a decision Fully agree Agree Do not agree/disagree Disagree Fully disagree Missing	50 (24) 126 (60) 22 (11) 8 (4) 4 (2) 0	31 (17) 114 (63) 25 (14) 8 (4) 2 (1) 5	38 (18) 114 (55) 32 (15) 18 (9) 7 (3) 5	40 (22) 94 (53) 22 (12) 20 (11) 4 (2) 10
8. I feel a responsibility towards those close to me to choose radiotherapy/extra radiotherapy (boost) Fully agree Agree Do not agree/disagree Disagree Fully disagree Missing	18 (9) 52 (25) 45 (21) 69 (33) 26 (12) 0	12 (7) 26 (14) 37 (20) 75 (41) 32 (18) 3	22 (11) 39 (19) 52 (25) 69 (33) 27 (13) 5	12 (7) 31 (17) 35 (20) 69 (39) 32 (18) 10
9. Reasons other than those mentioned above were the most important to me in making my decision Fully agree Agree Do not agree/disagree Disagree Fully disagree Missing	17 (8) 50 (24) 59 (29) 57 (27) 24 (12) 3	9 (5) 44 (24) 60 (33) 46 (26) 21 (12) 5	20 (9) 47 (22) 63 (30) 60 (28) 17 (8) 7	12 (7) 42 (24) 56 (31) 51 (29) 17 (10) 11

**Table:** 

	T1			
Question	Control (%)	Order*	Intervention (%)	Order*
1. I think the idea of radiation is really awful	36 (6)	7	25 (5)	7/8
2. I’ll do whatever it takes to reduce the chances of tumor recurring	138 (23)	1	94 (19)	2
3. I’m put off by the idea of daily radiation treatment/extra days of radiotherapy (boost)	28 (5)	8	16 (3)	9
4. I’m put off by the radiation side effects	71 (12)	4	74 (15)	3
5. The cosmetic result in the long term is important to me	24 (4)	9	25 (5)	7/8
6. Choosing for radiotherapy/extra radiotherapy (boost) gives me peace of mind	81 (14)	3	69 (14)	4
7. What my health care provider advises is important to make a decision	124 (21)	2	102 (21)	1
8. I feel a responsibility towards those close to me to choose radiotherapy/extra radiotherapy (boost)	45 (8)	5	34 (7)	6
9. Reasons other than those mentioned above were the most important to me in making my decision	44 (7)	6	47 (10)	5
Total	591		486	

	T2			
	Control (%)	Order*	Intervention (%)	Order*
1. I think the idea of radiation is really awful	26 (5)	8	32 (7)	6
2. I’ll do whatever it takes to reduce the chances of tumor recurring	132 (24)	1	100 (20)	1
3. I’m put off by the idea of daily radiation treatment/extra days of radiotherapy (boost)	30 (5)	7	20 (4)	8
4. I’m put off by the radiation side effects	58 (10)	4	72 (15)	3
5. The cosmetic result in the long term is important to me	14 (2)	9	19 (4)	9
6. Choosing for radiotherapy/extra radiotherapy (boost) gives me peace of mind	92 (16)	3	71 (14)	4
7. What my health care provider advises is important to make a decision	117 (21)	2	97 (20)	2
8. I feel a responsibility towards those close to me to choose radiotherapy/extra radiotherapy (boost)	45 (8)	6	31 (6)	7
9. Reasons other than those mentioned above were the most important to me in making my decision	47 (8)	5	50 (10)	5
Total	561		492	

## Appendix F . Patient preferences, as indicated in the self-developed questionnaire. These statements are similar to the statements asked for in the PtDA, which was developed together with patients. T1 = < 3 days after the decision, T2 is 3 months after the decision

Can you identify which 3 aspects from the 9 mentioned above were the most important for you when making the choice to undergo radiotherapy or not? (patients could fill in 3 boxes on the form).*Order of most frequent to least frequent chosen answer.
